# Effect of Catgut Embedment in Du Meridian Acupoint on Mental and Psychological Conditions of Patients with Gastroesophageal Reflux Disease

**DOI:** 10.1155/2020/5415813

**Published:** 2020-09-22

**Authors:** Zhengjie Luo, Xuanming Hu, Chaoming Chen, Lvqun Zhu, Wenyan Zhang, You Shen, Jirou He

**Affiliations:** ^1^Department of Rehabilitation Medicine, Third Affiliated Hospital of Soochow University & Changzhou First People's Hospital, Changzhou 213003, Jiangsu, China; ^2^Department of Acupuncture, Nanjing Hospital of Chinese Medicine, Affiliated to Nanjing University of Chinese Medicine, Nanjing 210001, Jiangsu, China; ^3^Nanjing University of Chinese Medicine, Nanjing 210029, Jiangsu, China

## Abstract

**Objective:**

To observe the influence of the catgut-embedding method in Du Meridian acupoint on the mental and psychological state of patients with gastroesophageal reflux disease (GERD) and analyze its possible mechanism.

**Methods:**

According to the random number table, 60 patients with GERD were randomly divided into groups of acupoint catgut embedding and Western medicine, 30 cases in each group. The acupoint group was given catgut embedment in the positive reaction points along the Du Meridian, while the Western medicine group received lansoprazole tablet. They were both treated for six weeks. Scores of Gastroesophageal Reflux Disease Questionnaire (GerdQ), Zung's Self-Rating Anxiety Scale (SAS), Zung's Self-Rating Depression Scale (SDS), and Health-Related Quality of Life Scale for GERD (GERD-HRQL) were measured before and after treatment to analyze and evaluate the differences of symptom scores and mental and psychological conditions between the two groups.

**Results:**

(1) The scores of GerdQ, GERD-HRQL, SAS, and SDS in the two groups both significantly decreased after treatment (*P* < 0.05), and those of the acupoint group were much lower than the Western medicine group (*P* < 0.05). (2) The total effective rate was 90.00% in the acupoint group and 53.33% in the Western medicine group, with a statistically significant difference (*P* < 0.05). (3) The correlation coefficients *r* between the GerdQ score and scores of SAS and SDS were 0.563 and 0.322, respectively, and those between the GERD-HRQL score and scores of SAS and SDS were, respectively, 0.506 and 0.435.

**Conclusion:**

(1) The main symptoms of GERD patients, such as acid reflux and heartburn, mental and psychological condition, and quality of life, were all improved in the two groups, but the efficacy in the acupoint group is superior to that of the Western medicine group. (2) The clinical symptoms and scores of patients' quality of life are positively correlated with the degree of their anxiety and depression. (3) The acupoint catgut-embedding method can effectively regulate the anxiety and depressive symptoms of patients, which complements the efficacy of proton-pump inhibitors and benefits a wider range of population.

## 1. Introduction

The mental and psychological factors can affect the pathogenesis, diagnosis, and prognosis of gastroesophageal reflux disease (GERD) [1]. They are the important causes of high sensitivity of esophagus viscera, which stimulate the brain to generate signals that travel down to the esophagus and gastrointestinal tract, causing gastrointestinal reactions and abnormal gastric acid secretion and worsening the symptoms of the digestive tract. Long-term suffering of clinical symptoms, in turn, can aggravate patients' anxiety and depression, leading to autonomic nerve dysfunction. The imbalance between sympathetic and parasympathetic nerves reduces the contraction force of digestive tract circular fold muscle, which further slows down gastric contraction rate, decreases migrating motor complex (MMC) function, and regurgitates stomach acid to the esophagus, eventually generating the reflux esophagitis [2, 3]. Current studies have shown that acupuncture has an accurate therapeutic effect on both the gastrointestinal somatization symptoms caused by psychosocial diseases and the psychosocial symptoms generated together with gastrointestinal diseases, and its efficacy is better than Western medicine in improving some symptoms [4, 5]. The catgut-embedding therapy, an important part of external treatment methods of traditional Chinese medicine, stimulates acupoint with a longer effect than simple acupuncture. It is simple and safe in operation, which can balance *yin* and *yang*, dredge *qi* and blood by stimulating acupoint based on syndrome differentiation of meridians and collaterals so as to reduce symptoms and improve patients' quality of life. The previous study of our research group has proved that acupuncture and catgut embedment in Du Meridian acupoint have better effects on GERD than oral administration of proton-pump inhibitor (PPI) [6, 7]. On this basis, this study discussed the influence of Du Meridian acupoint catgut embedding on the psychosocial factors of GERD, objectively evaluated its therapeutic effect on the disease, and analyzed its mechanism of improving patients' psychosocial symptoms, in order to support the application and promotion of Du Meridian catgut-embedding therapy in the future.

## 2. Clinical Data

### 2.1. General Information

This study was approved by the Hospital Ethics Committee. In this study, 60 patients diagnosed with GERD in the Acupuncture and Moxibustion Department of Nanjing Hospital of Chinese Medicine, the Acupuncture, Moxibustion and Tuina Department of Jiangdong Community Health Service Center, and the National TCM Physician Outpatient Department affiliated to Nanjing University of Chinese Medicine from March 2018 to March 2019 were enrolled, and they or their immediate family members have signed the informed consent. According to the random number table, the subjects were randomly divided into the acupoint catgut-embedding group (30 cases) and the Western medicine group (30 cases). There were 14 males and 16 females in the acupoint group, aged 23–75 years and averaged 50.77 ± 15.73, with a disease duration of 0 to 30 years, averaged 5.42 ± 6.31. In the Western medicine group, there were 11 males and 19 females, aged 19–72 years and averaged 47.30 ± 14.83, with a disease duration of 0 to 20 years, averaged 4.42 ± 5.40. The differences in gender, age, and course of disease between the two groups had no systematic significance (*P* > 0.05) and were comparable. Among the 60 included GERD patients, 5 were identified by electronic gastroscopy as Barrett's esophagus (BE), 22 were identified as erosive esophagitis (EE), 20 were chronic gastritis, and the rest 10 and 3 cases were identified as chronic superficial gastritis and chronic atrophic gastritis, respectively.

### 2.2. Diagnostic Criteria

It refers to *the Montreal definition and classification of gastroesophageal reflux disease* (2006) [8] and the *Guidelines for the diagnosis and management of gastroesophageal reflux disease* (2013) [9]: (1) symptoms of heartburn, gastric acid reflux, noncardiac chest pain, and upper abdominal pain occurred for more than 4 weeks. (2) Erosive esophagitis or Barrett's esophagus is found by endoscopic examination. (3) For patients with obvious symptoms but having no organic changes under the gastroscope, if the symptoms were alleviated after oral administration of proton-pump inhibitor (PPI), they will be confirmed with GERD.

### 2.3. Inclusion Criteria

Inclusion criteria include (1) people showing in consistence with the diagnostic criteria; (2) age from 18 to 75 years; (3) at least one of the four typical GERD symptoms occurring with a duration longer than 4 weeks; and (4) those who voluntarily signed the informed consent and actively cooperate with the treatment.

### 2.4. Exclusion Criteria

Exclusion criteria include (1) patients outside the inclusion criteria; (2) patients accompanied by gastrointestinal bleeding or intestinal metaplasia under gastroscope; (3) acute abdominal patients with rebound pain according to abdominal examination; (4) those with connective tissue diseases and blood system diseases; (5) those intolerant to endoscopic examination; (6) pregnant and/or lactating women; and (7) those with severe hemorrhagic diseases.

### 2.5. Reasons for Loss of Contact and Corresponding Measures

These include the patients dropping out of the study or abandoning treatment during the study. The solution was to supplement the patient participants into the research group in proportion to 1 : 1.

### 2.6. Complying with the Study Principle of Blindness

The researchers were divided into four groups: the first group randomly assigned subjects; the second was responsible for performing the treatment; the third collected data; and the fourth was responsible for data collation, statistical analysis, and article writing. The four groups separately implemented their tasks.

## 3. Treatment Methods

### 3.1. Western Medicine Group

The patients were given lansoprazole tablets (Keyilin, 15 mg × 14 tablets, produced by Sichuan Hairong Pharmaceutical Co., Ltd., National Drug Approval Code H20065186), oral administration, 1-2 tablets (15–30 mg) per day for 6 weeks.

### 3.2. Acupoint Group

Acupoint selection: Baihui (DU 20), Dazhui (DU 14), the positive response points to tenderness in the thoracic dorsal segment of Du Meridian (TDSDM). Exploration of positive tenderness points: before treatment, the patient lies in a prone position, fully exposing the back, and both upper limbs are relaxed and naturally placed at the sides of the body. The operator should stand on the right side of the patient and place the right index finger pulp in the depression under the spinous process in the middle of the patient's back. The patient is then examined from the depression under the T1 spinous process to T9 spinous process depression with continuous and uniform pressure. At the same time, the operator will ask the patient whether there is pain, sour and swelling, and other discomforts, and where the discomfort exists is marked as a positive point, while those without pain or unable to clearly tell are considered as negative points. If a patient has a history of lumbago and back pain, the operator should acquire the details of his or her medical history and perform a physical examination or imaging examination for further exclusion, to avoid making mistakes in the study results due to wrong positive points [10]. The positive points along the Du Meridian were selected for catgut embedment, together with Baihui (DU 20) and Dazhui (DU 14). If there were no positive response points, Zhiyang (DU 9), Lingtai (DU 10), Shendao (DU 11), Dazhui (DU 14), and Baihui (DU 20) would be used as the substitute.

**Figure 1:**
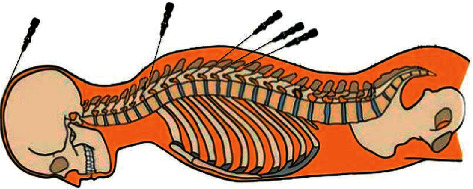
An example of the thread-embedding operation on the back.

Operation process (Figure 1): after cleaning and disinfecting the hands of the operator and the patient's fully exposed back, open the disposable bending plate, disposable embedding needle, absorbable protein thread, and metal tweezer and wear disposable sterile medical gloves. Then, pull out the embedding needle core about 2 cm and use the metal tweezer to place the protein thread into the needle tube from the needle tip. The operator held the embedding needle with one hand and lifted and pinched the skin on the back with the other hand to insert needles 0.1–0.3 inches from the acupoints selected at an angle of about 45° (between the needle body and the patient's back). After that, slowly push the needle inward until it entered the interspinous ligament in the gap between the spinous processes, slightly deeper than the length of the protein thread. Then, pull it back slightly to the subcutaneous soft tissues and perform twirling, lifting, and thrusting methods until the local muscles had obvious soreness, numbness, heaviness, and distension. Afterwards, push the needle core while pulling back the needle tube to embed the protein thread into the subcutaneous soft tissues. For thread embedment in Baihui (DU 20), the needle was inserted into the subgaleal loose connective tissues at an angle of 15–30° (between the needle body and the scalp). The protein thread should not be exposed outside the epidermis. Every time before thread embedding, it is necessary to check whether there was a nodule around Baihui (DU 20) caused by incomplete absorption of protein thread. If it exists, Baihui (DU 20) will not be used for thread embedment [10]. After pulling out the needles, the operator should press the needle holes immediately with sterile cotton balls for 5–10 minutes, covering sterile dressing and fixed with adhesive tape for 24 hours. The patient could not take a shower within 24 hours. The method was performed once every two weeks for a total of 3 times (Day 0, Weeks 2 and 4). The observation indexes were evaluated before the first treatment (Day 0) and on the 6^th^ week (Day 42) after the third treatment was finished.

## 4. Curative Effect Observation

### 4.1. Observation Indexes

The two groups both underwent assessment twice before and after the treatment using scales of GerdQ, SDS, SAS, and GERD-HRQL for efficacy evaluation. The patients in the two groups were surveyed with a questionnaire before treatment (Day 0). The Western medicine group was treated with lansoprazole orally for 6 weeks and received the second assessment on the 42^nd^ day. The acupoint group underwent the second and third catgut-embedding treatment on the 14^th^ and 28^th^ days, respectively, and they also received the second assessment on the 42^nd^ day.

#### 4.1.1. GerdQ

Patients were asked to recall their symptoms for the last week to fill the GerdQ, including scoring of positive symptoms, negative symptoms, and positive impacts. The maximum cumulative score of the six questions was 18. The total score ≥8 supports the diagnosis of GERD. Scores of the three items were counted up to get the final score. The higher the score, the more serious the disease condition was.

#### 4.1.2. SDS

A total of 20 topics were included, using a 4-grade scoring method set according to the occurrence frequency of the main symptoms. The scores of all questions were added together and multiplied by 1.25, and the integer part was the standard score. The maximum score was 100, and over 53 indicated the existence of depression. The higher the score, the more serious the patient's depression was.

#### 4.1.3. SAS

A total of 20 topics were selected, using a 4-grade scoring method set according to the occurrence frequency of the main symptoms. The scores of all questions were added together and multiplied by 1.25, which was rounded to the nearest whole number to get the standard score. The maximum score was 100 and over 50 indicated the presence of anxiety. The higher the score, the more serious the patient's anxiety was.

#### 4.1.4. GERD-HRQL

Patients were scored with GERD-HRQL, including heartburn, dietary habits, dysphagia, flatulence, and medication, from 0 to 5 points by reference to the severity of those symptoms. The higher the score, the greater the impact on the patient's quality of life and the lower the quality of life.

### 4.2. Clinical Efficacy Determination

The effective rate of GerdQ score calculated by nimodipine scoring method: (a) cure: curative effect index = 100%; (b) marked effect: curative effect index ≥80%; (c) effective: curative effect index ≥50% and <80%; and (d) invalid: curative effect index <50%. Total effective rate = (number of cured cases + number of markedly effective cases + number of effective cases)/total number of cases × 100%.

### 4.3. Statistical Processing

In this study, IBM SPSS 23.0 was used for data collation and analysis. The measurement data were expressed as mean ± standard deviation, and the mean comparison of those with normal distribution between the two groups was evaluated by an independent sample *t*-test, while the comparison of those with nonnormal distribution was evaluated by rank-sum test. Pearson's correlation coefficient was used for correlation analysis. The count data was expressed by the case number. Chi-square test was used to compare the distribution of classification data among different groups. *Α* = 0.05 was considered as the statistical basis.

### 4.4. Results and Analysis

**Figure 2:**
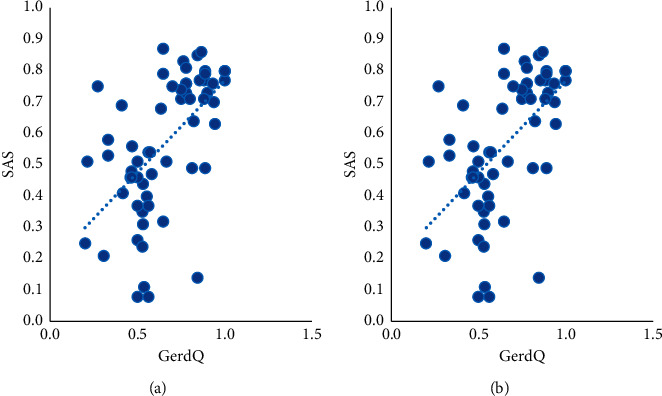
Scatter chart of correlation analysis between GerdQ score and scores of SAS and SDS.

**Figure 3:**
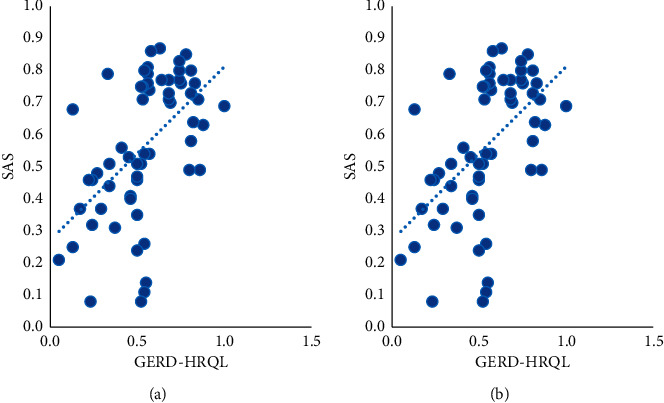
Scatter chart of correlation analysis between GERD-HRQL score and scores of SAS and SDS.

1. The results of electronic gastroscopy of 60 GERD patients showed that 27 cases had esophageal mucosal damage (5 cases of BE and 22 cases of EE), accounting for 45% while another 33 cases did not have (including 20 cases of chronic gastritis, 10 cases of chronic superficial gastritis, 3 cases of chronic atrophic gastritis). Before the clinical trial, patients without esophageal mucosal damage (accounting for 55%) showed significantly reduced reflux symptoms in the oral PPI testing, for which they were diagnosed as nonerosive reflux disease (NERD), as shown in Table 1.
2. The differences in scores of GerdQ, SAS, SDS, and GERD-HRQL of the two groups were not statistically significant before treatment (*P* > 0.05), and there was comparability between the two groups. After treatment, those scores in the two groups all decreased with a significant difference before and after treatment (*P* < 0.05), indicating that the treatment methods used in the two groups are both effective on GERD. The scores of symptoms and quality of life of the acupoint group after treatment were significantly lower than those of the Western medicine group (*P* < 0.05), suggesting that the effect in the acupoint group is superior to that of the Western medicine group, as shown in Table 2.
3. Correlation analysis was expressed by Pearson's correlation coefficient. The correlation coefficient *r* values of GerdQ score with scores of SAS and SDS were 0.563 and 0.322 respectively, and the *r* value of GERD-HRQL score with scores of SAS and SDS were, respectively, 0.506 and 0.435, suggesting that the clinical symptoms and quality of life of GERD patients were positively correlated with the degree of anxiety and depression, as shown in Table 3 and Figures 2 and 3.
4. Comparison of efficacy at the end of the treatment between the two groups using the chi-square test. The curative effect was 90.00% in the acupoint group and 53.33% in the Western medicine group after treatment, showing a statistically significant difference between the two groups. The rate of the marked effect and the total effective rate in the acupoint group were significantly higher than those of the Western medicine group (*P* < 0.05), as shown in Table 4.

## 5. Discussion

### 5.1. Relationship between Du Meridian and Anxiety and Depression

Du Meridian is closely related to the brain since it passes from the uterus to the top of the head along the posterior median line, as described in the Chapter “Yingqi” of *Huangdi Neijing: Lingshu*. The brain is the house of the original spirit and the center of consciousness and thought. According to TCM theories, meridians can be applied to treat diseases located along their running routes; thus, the acupoint of Du Meridian can be used to treat brain-related diseases, such as depression and other mental diseases. Du Meridian governs all other *yang* meridians (hand and foot tai*yang* meridians, hand and foot *shaoyang* meridians, and hand and foot *yangming* meridians); therefore, *yang qi* can supplement the brain and marrow by flowing through the running route of Du Meridian, providing the “material basis” the brain needs to maintain physiological functions. However, if *yang qi* cannot flow upward or the meridians and collaterals are obstructed, the *qi* flow of Du Meridian will be blocked and fail to transport essence to nourish the brain, which may lead to mental diseases such as depression and anxiety. Therefore, the smooth qi flow of Du Meridian is a key factor affecting the normal functions of brain governing mind and consciousness. In addition, since Du Meridian is connected with the bladder meridian of foot *taiyang*, the back-transport points (BL 13, BL 15, BL 18, BL 20, and BL 23) are closely related to the *zang-fu* organs (lungs, heart, liver, spleen, and kidney); the dysfunction of the *zang-fu* organs can lead to emotional dysfunction. In addition, the bladder meridian of foot *taiyang* is connected with Du Meridian through qi and blood, so the corresponding back-transport points or points of Du Meridian are suggested to treat depression and anxiety. They can also exert the effects of regulating functions of *zang-fu* organs related to emotional disorders, such as liver, heart, pericardium, spleen, and kidney (which are different from the organs in Western medicine). Moreover, since liver meridian of foot *jueyin* converges with Du Meridian at the top of head, acupoint of Du Meridian can also be used for dredging liver *qi*. It can also be applied to calm the mind.

Among patients with depression or gastrointestinal diseases, positive reaction points may occur in the TDSDM. Zhang and Wang [10] found that in patients with depression, the occurrence rate of tenderness reaction in the thoracic dorsal segment of Du Meridian reached over 80%. Moreover, the probability of positive points of tenderness occurring under the spinal process of T3 (DU 12) to T7 (DU 9) was the highest. According to clinical observation, this study found that the tenderness points in TDSDM of GERD patients were highly consistent with those of depression patients. The detection of these positive reaction points importantly guides the clinical diagnosis and treatment of depression and gastroesophageal reflux disease with acupuncture and moxibustion [4].

### 5.2. Discussion on the Mechanism of Acupoint Catgut-Embedding Regulating the Mental and Psychological State of GERD Patients

#### 5.2.1. Mechanism of Du Meridian Acupoint Catgut Embedding in the Treatment of GERD

Studies have shown that acupuncture can improve the loose lower esophageal sphincter (LES) of GERD patients by regulating the neuroendocrine-immune network, promote gastrointestinal motility, inhibit the secretion of gastric acid, and protect the gastric mucosa. It has a definite effect on the improvement of mental and psychological disorders such as depression and anxiety [11]. In terms of improving loose LES, acupuncture takes effect by enhancing its resting pressure and regulating gastrin and motilin to strengthen antireflux defense barrier [12, 13]. In terms of promoting gastrointestinal motility, acupuncture takes effect by regulating the level of the vasoactive intestinal peptide in digestive system diseases [14]. In terms of inhibiting gastric acid secretion and protecting gastric mucosa, acupuncture affects gastric acid secretion by regulating the changes of media concentration in the autonomic nervous system, gastrointestinal hormones, intestinal nervous system, and gastrointestinal mucosal tissues [15]. In terms of regulating psychological disorders, Pilkington [16] believes that acupuncture may play an antianxiety role by regulating signaling substances such as central serotonin, norepinephrine, dopaminergic, or hypothalamic-pituitary-adrenal axis (HPA axis) related hormones.

Acupoint catgut-embedment, an innovative extension of acupuncture treatment which broadens the application range of acupuncture, achieves the efficacy by harmonizing *yin* and *yang*, balancing functions of *zang-fu* organs, unblocking meridians and collaterals, regulating *qi* and blood, tonifying deficiency and purging the excess, and reinforcing healthy *qi* and dispelling pathogens, as well as taking advantage of the effects of needle retaining and catgut embedding. From the perspective of Western medicine, acupoint catgut-embedment can repair nerve functions and regulate nerve conduction and reflex, improve human immune function and local microcirculation, inhibit the production of inflammatory factors, reduce cell apoptosis, and regulate cytokines to accelerate metabolism [17]. It can not only exert the effects similar to that of the ordinary acupuncture but also promote continuous treatment by prolonging the stimulation duration, which is particularly suitable for multisystem chronic diseases. To some extent, it may reduce or replace the use of Western medicine. In addition to regulating the neuroendocrine-immune network, catgut-embedding therapy in acupoint of Du Meridian also treats GERD by improving the resting pressure of LES, preventing its transient loose, improving its ability to clear gastric acid, enhancing the resistance and repair function of gastroesophageal mucosa, accelerating the emptying of the gastrointestinal tract, promoting the gastrointestinal migrating motor complex (MMC), and reducing the sensitivity of human internal organs so as to improve its antireflux defense mechanism, thus inhibiting the occurrence of reflux.

#### 5.2.2. Mechanism of Catgut Embedment in Acupoint of Du Meridian on Mental and Psychological Regulation

Although there are no disease names of “depression” and “anxiety” in traditional Chinese medicine, they can be categorized into the range of “depression syndrome,” “visceral agitation (hysteria),” “lily disease,” and “insomnia” according to their characteristics. Acupuncture may regulate symptoms of anxiety and depression associated with digestive diseases by regulating the gut-brain axis, a network connecting the brain and the intestine through the autonomic nervous system and the neuroendocrine system of the HPA axis [18]. The gut-brain axis is basically composed of the central nervous system (CNS), autonomic nervous system (ANS), and enteric nervous system (ENS) [19]. Among them, CNS directly regulates gastrointestinal motility and secretion and ANS coordinates the brain and intestines through sympathetic and parasympathetic afferent and efferent neurons while ENS, mainly including the intercostal nerve and submucosal nerve plexus, plays an independent role in regulating intestinal movement and secretion [20]. The neuroendocrine network that links the three nervous systems to the brain is known as the gut-brain axis. This is how the brain interacts with the gut so as to regulate mood.

According to clinical observation, catgut embedment in Du Meridian acupoint may improve psychological disorders and relieve symptoms of anxiety and depression by regulating the dysfunctional gut-brain axis, thus adjusting the emotions of GERD patients. On the other hand, the catgut-embedding therapy in this study improved and cured symptoms of gastric acid reflux and heartburn, thus relieving symptoms of anxiety and depression and improving patients' psychological state and quality of life.

In conclusion, catgut-embedding therapy in acupoint of Du Meridian can effectively adjust the anxiety and depression symptoms, making up for the shortage of PPI and benefiting a wider range of patients. It can also reduce or replace the use of Western medicine for GERD patients accompanied by anxiety or depression and effectively improve physical and psychological symptoms.

## 6. Limitations and Prospects

There are some limitations in this clinical study. (1) With relatively small sample size, the scores of clinical symptoms and anxiety and depression scales may be easily affected by the subjective factors of patients. (2) This study lacks more objective evaluation indexes such as the comparison in esophageal dynamics and esophageal pH examination before and after treatment. (3) Limited by experiment conditions, the standard pressure measuring device was unavailable to determine the range and degree of the positive reaction points, which may cause errors due to the uneven press or overexertion of researchers.

24-hour pH monitoring of the esophagus is currently considered as one of the best examination methods and gold standards for GERD. There are certain limitations in this study due to diagnosing GERD solely through symptoms and electronic gastroscopy, without using the 24-hour esophagus pH monitoring restricted by conditions. In the following study, our team will explore the correlation of the results of 24-hour pH monitoring of the esophagus and symptoms with anxiety and depression in patients with GERD, and the effect of acupuncture treatment on pH values of the esophagus of GERD patients.

The symptoms of GERD are related to mild mental disorders (depression and anxiety). Although the results of this study show a direct improvement effect of acupoint catgut-embedding therapy on anxiety and depression of GERD patients, it is not convincing enough without setting a placebo group of acupuncture to eliminate the influence of subjective factors of patients. In the future, we will carry out further related studies to provide more evidence on the effects of acupoint catgut-embedding therapy on anxiety and depression of GERD patients.

Acupuncture and catgut-embedding therapy are effective methods to treat GERD. Further studies are needed to discuss the mechanism of how they take effects by regulating the gut-brain axis. Due to the limited time of clinical research, the levels of gastrointestinal hormones before and after catgut embedding were not monitored, and the relationship between them and the mental state was not discussed. In the later period, our research team will conduct research on the correlation between TDSDM and the gut-brain axis to explore the biological effect of catgut-embedding therapy on the neuroendocrine system of GERD patients, so as to provide a new theoretical basis for its clinical application in GERD.

## Figures and Tables

**Table 1:** Gastroscopy results of 60 patients.

	Barrett's esophagus (BE)	Erosive esophagitis (EE)	Chronic gastritis	Chronic superficial gastritis	Chronic atrophic gastritis
Total (60 cases)	5	22	20	10	3
Percentage	8.33	36.67	33.33	16.67	5

**Table 2:** Comparison of the two groups in different indicators before and after 6 weeks of treatment (*x*(−) ± *s*).

	The acupoint group	The Western medicine group	*t*	*P*
GerdQ-Day 0	14.77 ± 3.05	14.73 ± 2.05	0.050	0.961
GerdQ-6^th^ week	3.03 ± 2.46	6.93 ± 2.08	−6.632^*∗∗∗*^	0.000
*t*	16.464^*∗∗∗*^	15.625^*∗∗∗*^		
*P*	0.000	0.000		

GERD-HRQL-Day 0	26.23 ± 5.75	25.80 ± 4.54	0.324	0.747
GERD-HRQL-6^th^ week	8.57 ± 4.38	14.80 ± 4.65	−5.347^*∗∗∗*^	0.000
*T*	15.140^*∗∗∗*^	11.863^*∗∗∗*^		
*P*	0.000	0.000		

SAS-Day 0	63.00 ± 8.16	63.10 ± 6.25	−0.053	0.958
SAS-6^th^ week	15.53 ± 3.78	38.50 ± 8.27	−13.831^*∗∗∗*^	0.000
*T*	27.366^*∗∗∗*^	12.606^*∗∗∗*^		
*P*	0.000	0.000		

SDS-Day 0	59.70 ± 6.08	59.53 ± 6.95	0.099	0.922
SDS-6^th^ week	32.10 ± 5.9	38.17 ± 8.27	−3.270^*∗∗*^	0.002
*T*	21.549^*∗∗∗*^	9.754^*∗∗∗*^		
*P*	0.000	0.000		

^*∗∗*^ *P* < 0.01; ^*∗∗∗*^*P* < 0.001.

**Table 3:** Correlation analysis of the scores in the two groups.

		GerdQ	GERD-HRQL	SAS
GERD-HRQL	*r*	0.532^*∗∗∗*^	1	
	*P*	0.000		

SAS	*r*	0.563^*∗∗∗*^	0.506^*∗∗∗*^	1
	*P*	0.000	0.000	

SDS	*r*	0.322^*∗*^	0.435^*∗∗*^	0.326^*∗*^
	*P*	0.012	0.001	0.011

^*∗*^ *P* < 0.05; ^*∗∗*^*P* < 0.01; ^*∗∗∗*^*P* < 0.001.

**Table 4:** Comparison of clinical efficacy between the two groups.

	Markedly effective (case)	Effective (case)	Invalid (case)	Rate of marked effect (%)	Total effective rate (%)
The acupoint group	15	12	3	50.00	90.00
The Western medicine group	3	13	14	10.00	53.33
Statistical value	11.429	0.069	9.932	11.429	9.932
*P*	0.001	1.000	0.003	0.001	0.003

## Data Availability

The data used to support the findings of this study are included within the article.
